# miR-153 suppresses IDO1 expression and enhances CAR T cell immunotherapy

**DOI:** 10.1186/s13045-018-0600-x

**Published:** 2018-04-23

**Authors:** Qian Huang, Jiajia Xia, Lei Wang, Xu Wang, Xiaodong Ma, Qipan Deng, Yong Lu, Munish Kumar, Zhiyuan Zhou, Ling Li, Zhaoyang Zeng, Ken H. Young, Qing Yi, Mingzhi Zhang, Yong Li

**Affiliations:** 1 Department of Cancer Biology, Lerner Research Institute, Cleveland Clinic, Cleveland, OH 44195 USA; 2 Institute for Brain Research and Rehabilitation, South China Normal University, Guangzhou, 510631 China; 3 Raman Fellow (UGC), Department of Biochemistry, University of Allahabad, Allahabad, India; 4 Department of Oncology, The First Affiliated Hospital of Zhengzhou University, Lymphoma Diagnosis and Treatment Center of Henan Province, Zhengzhou, 450000 Henan Province China; 5 Cancer Research Institute, Central South University, Changsha, 410078 China; 6 Department of Hematopathology, The University of Texas M.D. Anderson Cancer Center, Houston, TX 77030 USA

**Keywords:** Chimeric antigen receptor (CAR), T cells, Indoleamine 2,3-dioxygenase 1 (IDO1), miR-153, Colon cancer, Tumor microenvironment

## Abstract

**Background:**

Indoleamine 2,3-dioxygenase 1 (IDO1) catalyzes the first and rate-limiting step in converting tryptophan to kynurenine. Chimeric antigen receptor (CAR) T cells are T cells with recombinant receptors targeting tumor-associated antigens. The Food and Drug Administration has approved CAR T cells that target CD19 for treatment of advanced B cell leukemia and lymphoma. However, CAR T cell therapy in solid tumors has been hampered by multiple obstacles. Preclinical and clinical studies suggest that combinatorial immune checkpoint blockade and IDO1 inhibition provide durable therapeutic efficacy against cancer. Yet, the combination of IDO1 inhibition and CAR T has not been attempted.

**Methods:**

We analyze IDO1 downregulation by miR-153 in colon cancer cells and the association of IDO1 and miR-153 expression with colorectal patient survival. We generate CAR T cells targeting the epidermal growth factor receptor variant III and measure their tumor killing effects against colon cancer cells with or without miR-153 overexpression by killing assays and in xenografts.

**Results:**

IDO1 is highly expressed in colorectal tumors and is inversely associated with patient survival. miR-153 directly inhibits IDO1 expression by targeting its 3′ untranslated region in colon cancer cells; yet, miR-153 overexpression does not affect cancer cell survival, apoptosis, and colony formation. When colon cancer cells are targeted by CAR T cells, miR-153 overexpression within tumor cells significantly enhances T cell killing in vitro and suppresses xenograft tumor growth in mice.

**Conclusions:**

These findings indicate that miR-153 inhibits IDO1 expression in colon cancer cells and is a tumor-suppressive miRNA that enhances CAR T cell immunotherapy. This study supports the combinatorial use of IDO1 inhibitors and CAR T cells in treating solid tumors.

**Electronic supplementary material:**

The online version of this article (10.1186/s13045-018-0600-x) contains supplementary material, which is available to authorized users.

## Background

Two major advances in immunotherapy tip the balance in favor of the immune system for eliminating cancer cells. First, in adoptive cell therapy [1], T cells from a cancer patient are subjected to gene transduction with chimeric antigen receptors (CARs) consisting of a tumor antigen-specific, single-chain variable fragment (scFv), and cytoplasmic tails containing co-stimulatory molecules; they are then infused back into the patient to attack and eliminate tumors. CARs act to redirect T cells’ effector functions, overcoming some limitations of the natural T cells such as the need for major histocompatibility complex (MHC) expression, MHC identity, and co-stimulatory signal. CAR T cells mediate higher levels of tumor-targeting activity [2, 3]. Patients with B cell malignancies have been effectively treated with CAR T cells specific for CD19, a pan-B cell marker. On August 30, 2017, the US Food and Drug Administration (FDA) approved Kymriah (tisagenlecleucel; CTL019) suspension for intravenous infusion, the first CAR T therapy, for the treatment of patients up to 25 years of age with B cell precursor acute lymphoblastic leukemia (ALL) that is refractory or in second or later relapse. Less than 2 months later, the FDA approved the second CD19 CAR T cell therapy (Yescarta (axicabtagene ciloleucel)) to treat adult patients with certain types of large B cell lymphoma who have not responded to or who have experienced relapse after at least two other kinds of treatment. Second, in checkpoint blockade therapy [2], antibodies specific to immune checkpoint inhibitory receptors like programmed death-1 (PD-1) and cytotoxic T lymphocyte antigen-4 (CTLA-4), or specific to their ligands, are infused into patients to enhance T cell killing of tumor cells. Numerous antibodies against PD-1 and CTLA-1 has been approved to treat multiple types of advanced solid tumors, including melanoma, non-small cell lung cancer, kidney cancer, bladder cancer, head and neck cancers, and Hodgkin lymphoma.

Despite its success in treating B cell malignancies, CAR T cell therapy for solid tumors has significantly lagged. The complex and heterogeneous tumor microenvironment of solid tumors plays an essential role in immunotherapeutic resistance, including preventing T cell proliferation and persistence and limiting T cell trafficking to tumor cells. Additionally, within the hostile tumor microenvironment, anti-tumor T cells encounter a number of challenges: suppressive cells such as T regulatory cells, myeloid-derived suppressor cells, tumor-associated macrophages, or neutrophils; suppressive soluble factors and cytokines; T cell-intrinsic negative regulatory mechanisms (e.g., upregulation of cytoplasmic and surface inhibitory receptors) and overexpression of inhibitory molecules (e.g., PD-L1 and PD-1 from tumor cells and infiltrating lymphocytes); acidic pH, hypoxia, and oxidative stress; and finally, specific to this study, nutritional depletion. Depletion of tryptophan and accumulation of its metabolites induce effector T cell apoptosis and dysfunction [4].

Indoleamine 2,3-dioxygenase 1 (IDO1), IDO2, and tryptophan 2, 3-dioxygenase (TDO) are the initial and rate-limiting enzymes of the tryptophan metabolism pathway [5, 6]. These enzymes convert tryptophan into kynurenine and 3-hydroxyanthranilic acid. Tryptophan is an essential amino acid for dendritic cells and T cell survival, proliferation, and activation. IDO1 is reported to be highly expressed in a wide range of cancers and IDO1 expression correlates with worse prognosis and reduced T cell infiltration [7–9]. IFN-γ is a crucial anti-tumor immune factor produced by cytotoxic T cells to induce MHC-I expression and enhance antigen presentation in tumor cells, which subsequently become more susceptible to MHC-restricted cytolytic killing [10]. However, cancer cells express higher levels of immunosuppressive molecules such as PD-L1 and IDO upon IFN-γ induction and thereby escape immune elimination [11]. Here, we show that IDO1 is significantly induced by IFN-γ treatment in colon cancer cells. By analyzing gene expression and patient data from The Cancer Genome Atlas (TCGA) database, we found that IDO1 overexpression inversely correlated with survival for patients with colon cancer patient. miR-153, a reported tumor-suppressive miRNA [12], downregulated the expression of IDO1 in colon cancer cells. Strikingly, CAR T cells carrying a scFv targeting EGFR displayed significantly higher cytotoxic activities against colon cancer cells overexpressing miR-153 than the control. Our results support that miR-153 is an endogenous IDO1 negative regulator and an immunotherapy enhancer.

## Methods

### Reagents and antibodies

*mir*Vana® miR-153 mimic and miRNA Mimic Negative Control #1 (negative control), Lipofectamine RNAiMAX transfection reagents, and IFN-γ were purchased from ThermoFisher (Waltham, MA). Cell culture medium RPMI1640 and DMEM were supplied by Cleveland Clinic Cell Culture Core Facility. Flow antibodies anti-ERBB2 (FITC), anti-EGFR (APC), anti-PD-L1 (APC) were purchased from BioLegend (San Diego, CA), and anti-IDO1 (APC) was purchased from eBioscience (Santa Clara, CA). Western blot antibodies against PD-L1 and IDO1 were purchased from Cell Signal Technology (Danvers, MA). T cell isolation and monocyte-derived dendritic cell isolation kits were purchased from StemCell technology (Vancouver, Canada). Western blot reagents and PVDF membranes were purchased from BioRad (Hercules, CA).

### Cell lines

The human noncancerous colon cell line CCD-841 and colon cancer cell lines HCT-116, SW620, HT-29, and DLD-1 were purchased from the American Type Culture Collection (ATCC) (Manassas, VA). Immortalized lung cell line BEAS-2B and lung cancer cell lines A549 and H-1299, 293 T, HeLa, and blood cancer cell lines (Jurkat, K562, Daudi, and Raji) were also from ATCC. All cell lines were authenticated by short tandem repeat profiling and were tested for and found to be mycoplasma-free. All cells were cultured in media recommended by ATCC. The efficiency for transient transfection of miR-153 miRNA mimic or anti-miR-153 in 3 colon cancer cell lines ranged from ~ 40-70%.

### CAR plasmid construction and CAR T cell preparation

For the CAR lentiviral vector, we replaced the copGFP gene in pSIH-copGFP with a CAR-expressing cassette against a variant of EGFR. The variant III mutation of the epidermal growth factor receptor (EGFRvIII) leads an in-frame deletion of a portion of the extracellular domain, creating a neoepitope [13]. The scFv we used is from cetuximab, a humanized monoclonal antibody against EGFRvIII. The *K*_*D*_ for this humanized scFv is 101 nM for EGFRvIII and 872 nM for WT EGFR [13]; therefore, it can target colon cancer cells expressing WT EGFR, albeit at a low efficiency. The scFv is fused to a CD8α hinge and transmembrane domain, and the intracellular domains of human CD28, 4-1BB, and CD3ζ [13]. Isolated T cells were derived from leukapheresis products obtained from de-identified healthy donors at Gulf Coast Regional Blood Center (Houston, TX). T cells were stimulated with Dynabeads Human T-Activator CD3/CD28 (ThermoFisher) at a bead-to-cell ratio of 3:1. T cells were then transduced by lentivirus carrying the CAR or the parental vector. The recombinant T cells were named CAR T or wild-type (WT) T and were expanded as described [13]. To detect CAR expression, we labeled recombinant T cells with Alexa Fluor® 647 AffiniPure Fab Fragment Donkey Anti-Mouse IgG (Jackson ImmunoResearch Laboratories, #715-607-003) and performed flow cytometry.

### Luciferase-based cytotoxicity assay and caspase 3/7-based apoptosis assay

DLD-1-luc and HCT-116-luc tumor cells were generated and employed in a modified version of a luciferase-based cytotoxicity assay [14]. Briefly, lentivirus carrying the firefly luciferase (*luc*) gene was transduced into DLD-1 or HCT-116 cell lines, followed by puromycin selection. The resulting recombinant DLD-1-luc or HCT-116-luc cells were resuspended at 2 × 10^4^ cells per well in 96-well plates and co-cultured with naive T cells or CAR T cells at varying ratios. After overnight incubation, luciferase substrates were added, and the relative luminescence units (RLU) were determined. Percent killing was calculated as 100 − 100 × [(RLU from wells with T cells and target cells)/(RLU from wells with target cells only)]. To analyze cellular apoptosis under T cell killing, we used the IncuCyte Caspase-3/7 Green Apoptosis Assay from Essen Bioscience (Ann Arbor, MI). DLD-1 or HCT-116 cells were co-cultured with varying amounts of T cells in the presence of NucView 488-labeled caspase 3/7 substrate (Essen Bioscience). After 24 h, green fluorescent (free NucView 488) signals were measured and used as a marker for dead cells. For the tumor cell apoptosis assay, cells were resuspended in a buffer containing annexin V (APC) and propidium iodide (PI) (BioLegend, San Diego, CA) and incubated for 10–15 min at room temperature before flow cytometry. For cell cycle analyses, cells were fixed with 70% ethanol for 30 min, then incubated with 50 μg/ml PI and 100 μg/ml RNase A at room temperature for 15 min. Stained cells were measured using a FACS-Calibur instrument (BD Biosciences, San Jose, CA) and analyzed with Cell-Quest 3.3 software. To analyze the expression of PD-L1 and EGFR, cells were stained with fluorochrome-labeled antibodies for 15 min before being subjected to flow cytometry. All flow cytometry data were analyzed using FlowJo 10 (FLOWJO, LLC; Ashland, OR).

### Other cellular assays

For ELISA, target tumor cells were co-cultured with T cells overnight at 37 °C, and supernatants were harvested and subjected to ELISA for cytokine production according to the manufacturer’s instructions (eBioscience). For the soft-agar assay [15], 5000 viable cancer cells were seeded in 1.5 mL tissue culture medium with 1% glutamine on top of 0.4% soft agar layered onto 0.8% solidified agar with tissue culture medium in 6-well plates. After incubation for 15 days, the colony foci were counted under a microscope. For the wound-healing assay [16], cancer cells were transfected as indicated and cultured for 24 h and then starved in culture medium containing 0.2% FBS for 12 h; the monolayer of confluent cells was scratched using a 10-μL pipette tip and cells were then photographed at different time points under an Olympus IX81 microscope. The relative wound areas were measured using ImageJ software (NIH, Bethesda, MD). When DLD-1 tumor cells were co-cultured with EGFRvIII-CAR T cells, the medium was first preconditioned by the tumor cells for 5 days.

### Xenografts in mice

NSG mice were purchased from the Jackson Laboratory (Bar Harbor, ME) and were housed under standard housing conditions at the animal facilities in the Cleveland Clinic Lerner Research Institute. The precursor to miR-153 (Pre-miR-153) sequence was inserted into a lentiviral vector pSIH-copGFP from System Biosciences (Palo Alto, CA) to generate pSIH-miR-153 [17]. DLD-1-luc cells were infected by lentivirus carrying miR-153, and 5 × 10^6^ recombinant cells (DLD-1 + miR-153) were subcutaneously injected into 6- to 8-week NSG mice (*n* = 5). As a control for DLD-1-miR-153 (DLD + NC), DLD-1-luc cells were infected with virus carrying the parental vector. After 7 days, 2 × 10^6^ EGFRvIII-CAR T cells or control T cells were injected through the tail vein. For bioluminescence imaging, mice were given luciferin 5 min before anesthesia (3% isoflurane), and imaging was performed and analyzed using the IVIS Spectrum In Vivo Imaging System (Perkin Elmer, Waltham, MA). All animal experimental procedures were approved by the Institutional Animal Care and Use Committee of the Cleveland Clinic.

### Statistical analysis

The data were analyzed with GraphPad (GraphPad Prism, version 5.02, La Jolla, CA). An unpaired two-tailed Student’s *t* test was performed for two-group comparisons. One-way analysis of variance was performed for multiple group comparisons with one independent variable. For patient survival, Kaplan-Meier analysis was followed by a log-rank Mantel-Cox test. Significance was set at *P ≤ 0.05, **P ≤ 0.01, and ***P ≤ 0.001. The Cancer Genome Atlas (TCGA) data were downloaded from TCGA portal (https://portal.gdc.cancer.gov/) or OncoLnc (http://www.oncolnc.org/).

## Results

### IDO1 induction by IFN-γ in cancer cells

**Fig. 1:**
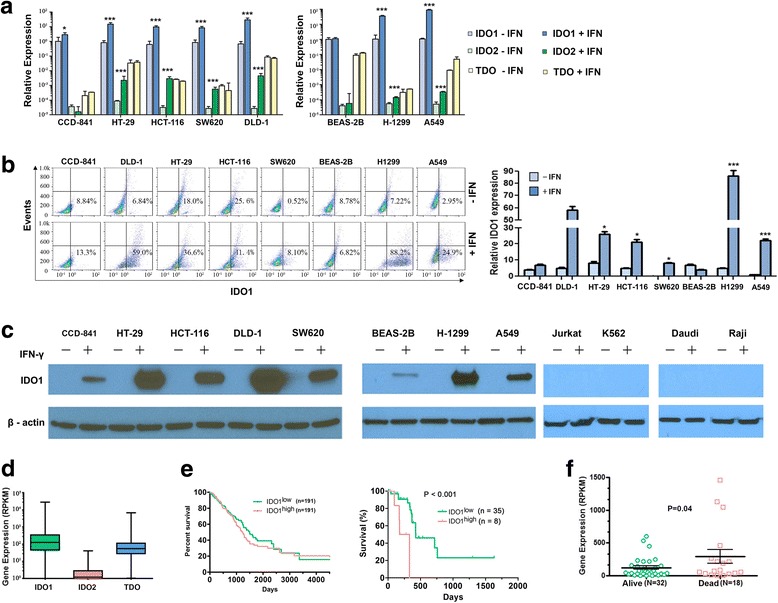
IDO1 expression in colon cancer cells and colon tumors. **a** IDO mRNAs were induced by IFN-γ in colon and lung cancer cells. The mRNA levels of IDO1, IDO2, and TDO were measured using qPCR. Cells were treated with 30 ng/ml IFN-γ for 24 h. **b** IDO1 protein expression measured by flow cytometry. *X*-axis, relative signal density of APC-labeled IDO1 antibodies; *Y*-axis, forward scattered light (i.e., events that indicate the number of cells). The panel on the right shows results (mean ± SEM) from three independent experiments. **c** Western blot analysis of IDO expression in colon and lung cancer cell lines and lymphoma cell lines. **d** RNA-Seq analysis of IDO1, IDO2, and TDO levels in 434 patients with colon cancer listed in the TCGA database. RPKM reads per kilobase of transcript per million mapped reads. IDO1 vs IDO2, *P* ≤ 0.001. IDO2 vs TDO, *P* ≤ 0.05. **e** Gene expression (mRNA) of IDO1 in relationship to survival in 382 patients with colon cancer (left) or in stage 2 patients only (right) in the TCGA database. The median IDO1 level was used to classify patients into the low and high groups, and statistical analysis was performed using log-rank Mantel-Cox test. **f** IDO1 expression in 50 patients with stage 2 colon cancer (same as **e** in the right side). Student’s *t* test was used to assess the difference between groups. *P ≤ 0.05; ***P ≤ 0.001

We determined the expression of IDO1, IDO2, and TDO in cancer cells treated with IFN-γ. We first used various concentrations (1 to 300 ng/ml) of IFN-γ to treat DLD-1 and A549 cells for 3 to 48 h and found in both cell lines that at 30 ng/ml IFN-γ, IDO1 expression rose to its highest level and plateaued at 24 h treatment as determined by flow cytometry (Additional file 1: Figure S1). We next treated the remaining cell lines with 30 ng/ml IFN-γ for 24 h and determined the mRNA levels of IDO1, IDO2, and TDO using quantitative real-time PCR (qPCR). As shown in Fig. 1a, IFN-γ upregulated IDO1 in all four colon cancer cell lines and two lung cancer cell lines, as well as in noncancerous colon epithelial CCD-841 cells, but not in immortalized lung epithelial BEAS-2B cells. IDO2 was significantly upregulated by IFN-γ in all six cancer cell lines (HT-29, HCT-116, SW620, DLD-1, H-1299, and A549), whereas TDO was upregulated in only A549 cells. Flow cytometry analyses validated IDO1 upregulation in these cancer cell lines (Fig. 1b). As shown on western blotting in Fig. 1c, IFN-γ treatment significantly elevated the IDO1 protein level in four colon cancer cell lines (HT-29, HCT-116, SW620, DLD-1) and two lung cancer cell lines (H-1299 and A549) and moderately increased IDO1 in CCD-841 and BEAS-2B cells. In contrast, IDO1 expression was undetectable in blood cancer cell lines (K562, Jurkat, Raji, and Daudi) with or without IFN-γ treatment.

### IDO expression in colon tumors

We analyzed IDO expression in tumors from colorectal cancer patient data in the TCGA. Based on RNA read numbers, IDO1 expression was much higher than IDO2 and TDO expression (Fig. 1d, *n* = 434 colorectal patients). From 382 patients with survival data, we found that the average overall survival was reduced by 5 months in IDO1^high^ patients compared with IDO1^low^ patients (*n* = 191 in each group, Fig. 1e, left panel); however, this was not statistically significant (*P* = 0.4). For patients with earlier stage cancer (stage 2; too fewer stage 1 patients to be included), lower IDO1 expression was associated with better survival (Fig. 1e, right panel, *P* < 0.001); IDO1 expression was much higher in those patients who had already died (*n* = 18, mean overall survival = 7 months) than in patients who were still alive (*n* = 32, mean overall survival = 10 months, Fig. 1f). However, no significant correlation between IDO2 or TDO gene expression levels and patient survival was found (Additional file 2: Figure S2).

### miR-153 downregulates IDO1 expression

**Fig. 2:**
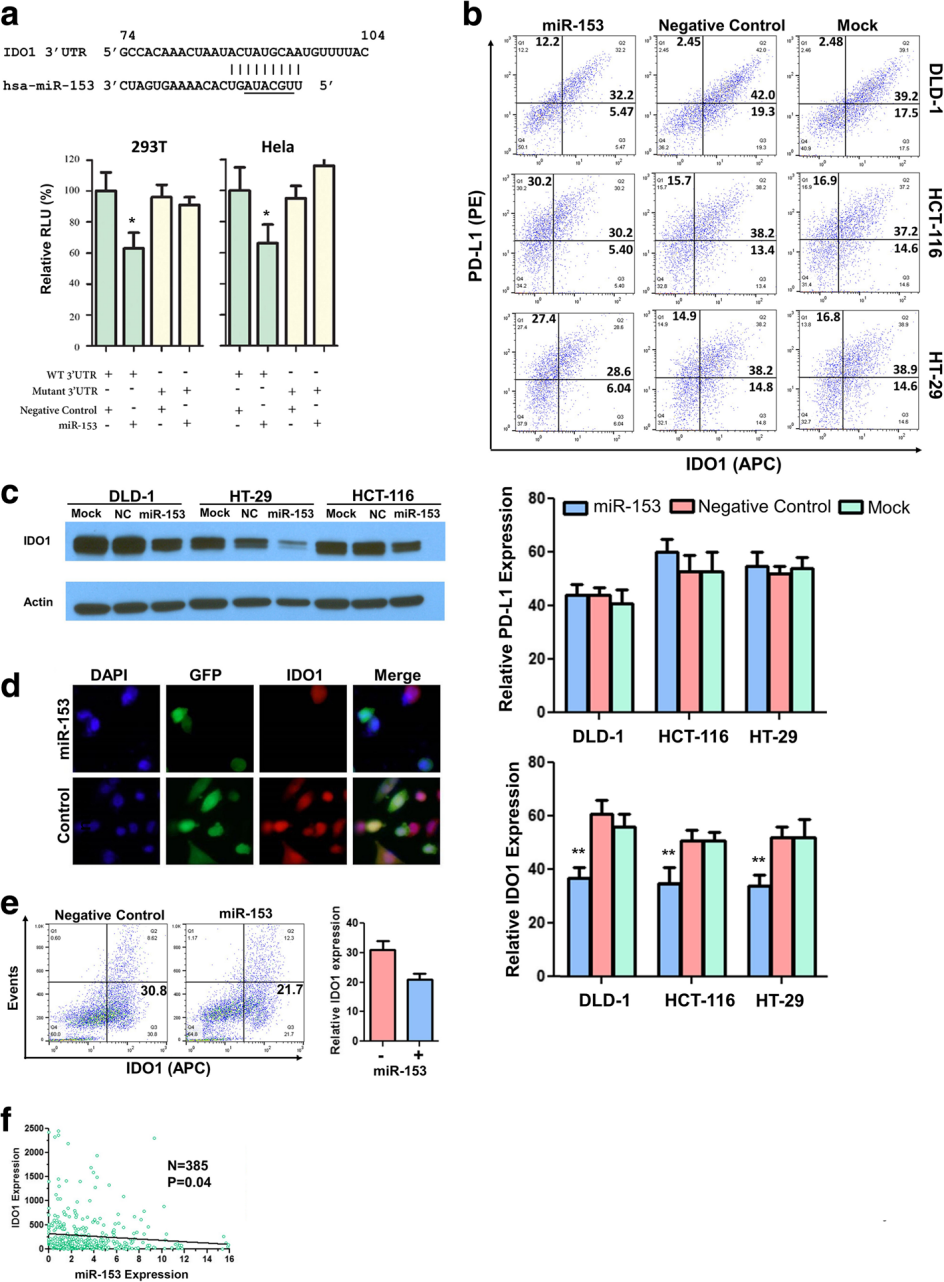
miR-153 downregulates IDO1 expression in colon cancer cells. **a** miR-153 inhibits reporter expression. At the top is the base pairing between IDO1 3′UTR and miR-153. The seed sequence of miR-153 is underlined. At the bottom is the reduced luciferase activity from reporter carrying the WT or the mutant IDO1 3′UTR under miR-153 overexpression. *Y*-axis denotes relative luciferase units. Dual luciferase reporter assays were performed three times from 293 T and Hela cells co-transfected with a *Renilla* luciferase gene (pRL-TK, Promega), a firefly luciferase gene (pGL-3Promoter) upstream of the WT (green) or the mutant (yellow) IDO1 3′UTR, and mirVana® miR-153 mimic (miR-153) and miRNA Mimic Negative Control #1 (NC). **b** Flow cytometry analyses of IDO1 and PD-L1 expression in DLD-1, HT-29, or HCT-116 cells transfected with miR-153, a negative control (NC), or mock treatment. **c** Western blotting analyses of IDO1 expression. **d** Immunofluorescence analyses of IDO1 protein expression (red) in DLD-1 cells expressing miR-153. The plasmids carrying miR-153 or the control had a GFP expression cassette. Nuclei were counterstained with DAPI (blue). **e** Flow cytometry analyses of IDO1 expression in monocyte-derived dendritic cells with miR-153 overexpression. A bar graph summarized fluorescence density (mean ± SEM) for IDO1 expression from three independent experiments (right). **f** Simple linear regression analysis showing an inverse relationship between miR-153 and IDO1 expression in 385 colon cancer patients from the TCGA database. *P ≤ 0.05; **P ≤ 0.01

We sought to study the regulation of IDO1 gene expression mediated by miRNAs. According to the computational program TargetScan version 7.1, which predicts biological targets of miRNAs by searching for the presence of conserved sites that match the seed region of each miRNA [18], the human *IDO1* gene is putatively targeted by miR-153 and other 9 miRNAs (Additional file 3: Figure S3A). We cloned the full-length IDO1 3′ untranslated region (3′UTR) downstream of the firefly luciferase 2 (*luc2*) gene in the pmirGLO vector. The pmirGLO vector constitutively expresses the *Renilla* luciferase gene as a reference reporter. We introduced these 10 miRNAs and pmirGLO-IDO1-3’UTR into 293 T or Hela cells. We found only miR-153 downregulated *luc2* expression in both 293 T and Hela cell lines (Additional file 3: Figure S3B and Fig. 2a). When the miR-153 binding site in the IDO1 3’UTR was mutated, the reporter downregulation by miR-153 was abolished (Fig. 2a).

We transfected miR-153 mimics or a negative control into colon cancer cell lines DLD-1, HCT-116, and HT-29 and treated the cells with IFN-γ. Compared with mock treatment (transfection without agents) or the negative control, miR-153 substantially decreased IFN-γ-induced IDO1 expression in all cell lines as determined by flow cytometry (Fig. 2b) and about 60–90% by western blotting (Fig. 2c). The difference between flow cytometry and western blotting was likely a result of methodological variances (western blotting measures total soluble proteins from a pool of cells; flow cytometry measures the fluorescent density of individual cells). We noted that PD-L1, another IFN-γ-induced protein, was not suppressed by miR-153 (Fig. 2b). To corroborate this finding, we next transfected a miR-153 expression vector containing both miR-153 and GFP into DLD-1 cells and stained cells using antibodies against IDO1. We found that the IDO1 staining signal was substantially reduced in the transfected GFP+ cells compared with the untransfected (GFP−) cells (Fig. 2d). miR-153 also decreased IDO1 expression in monocyte-derived dendritic cells (Fig. 2e). Next, we used LPS or Cp-G DNA instead of IFN-γ to treat DLD-1 cells and found that both LPS and Cp-G DNA induced IDO1 expression. Introduction of miR-153 resulted in lower IDO1 expression in DLD-1 cells treated with LPS or Cp-G DNA (Additional file 4: Figure S4A and B). IDO-1 downregulation by miR-153 was also observed in HCT-116 cells treated with LPS (Additional file 4: Figure S4C). miR-153 decreased IDO1 mRNA expression in DLD-1 cells, but not in HCT-116 cells (Additional file 4: Figure S4D). We noted that mammalian miRNAs downregulate gene expression through both mRNA degradation and/or translational repression [19]. Nonetheless, these data suggest that IDO1 is a bona fide target gene of miR-153. We analyzed miR-153 expression in relationship to IDO1 mRNA levels in patients with colorectal cancer and found a significant inverse correlation between miR-153 and IDO1 mRNA levels (*P* = 0.04, *R*^2^ = 0.23; Fig. 2f). However, patients’ survival was not associated with miR-153 expression levels.

### miR-153 overexpression has little effects on colon cancer cells

**Fig. 3:**
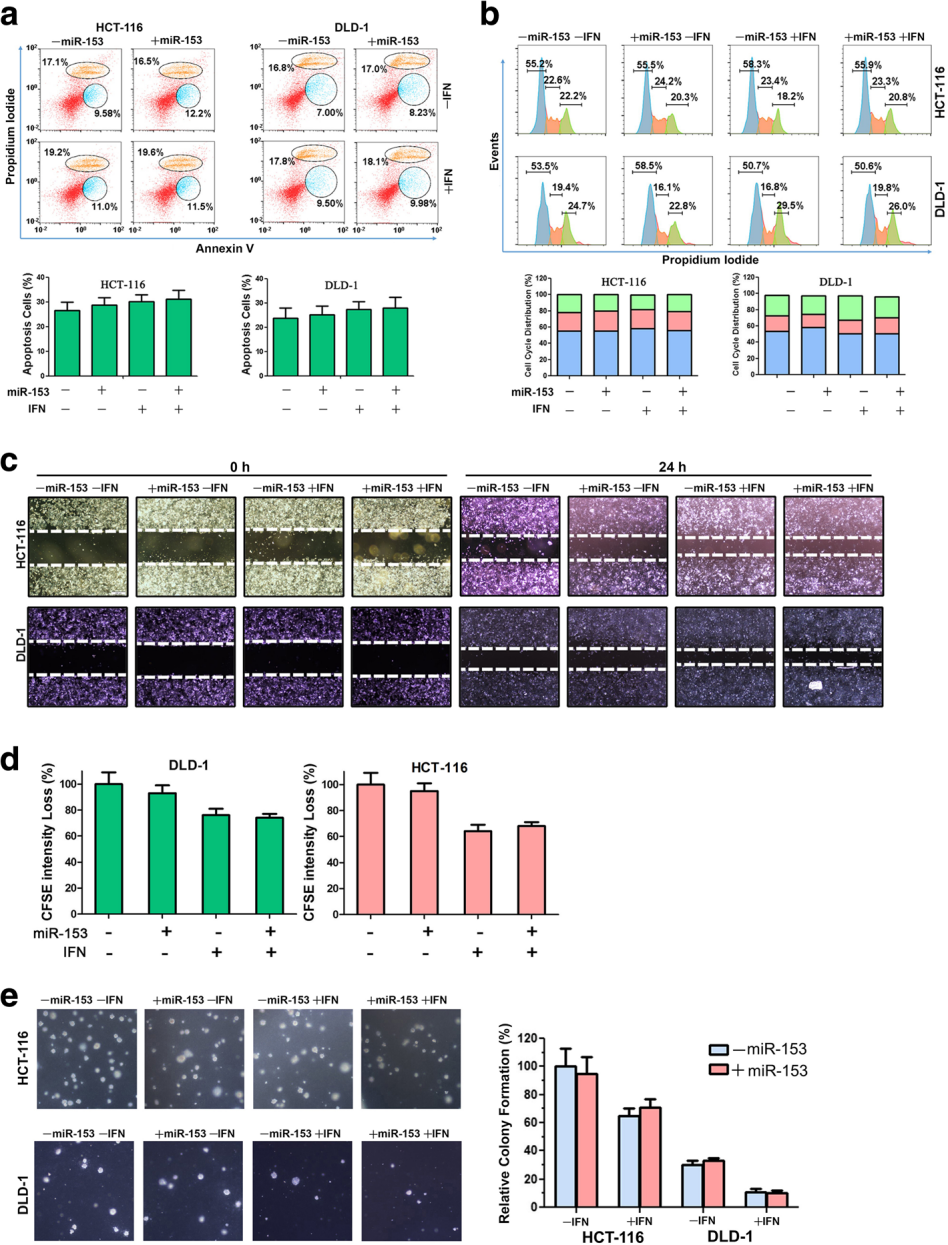
miR-153 overexpression alone has little effect on colon cancer cells. **a** Apoptosis analyses of DLD-1 and HCT-116 cells transfected with miR-153 and treated with IFN-γ. At the top are the representative images of flow cytometry; early apoptotic cells were in blue and late apoptotic cells in orange. At the bottom is the summary for the flow cytometry data. **b** Cell cycle analyses of DLD-1 and HCT-116 cells transfected with miR-153 and treated with IFN-γ. Blue is for G0/G1 phase, orange is for S phase, and green is for G2 phase. At the top are the representative images of flow cytometry. At the bottom is the summary for the flow cytometry data. **c** Wound-healing analyses of DLD-1 and HCT-116 cells transfected with miR-153 and treated with IFN-γ. Images were taken immediately after or 24 h after scratch. **d** Cell proliferation assays for DLD-1 and HCT-116 cells. CFSE-labeled cells were transfected with miR-153, treated with IFN-γ, and cultured for 1 to 6 days before flow cytometry. A bar graph was plotted to show loss of CFSE fluorescence intensity in cells with or without miR-153 overexpression treated with IFN-γ. **e** Colony formation assays of DLD-1 and HCT-116 cells transfected with miR-153 and treated with IFN-γ. In the left side is the representative images of colonies formed on soft agar. In the right side is the summarized data for relative colony numbers as determined from three independent experiments

We determined whether miR-153 affects colon cancer cell phenotypes through a series of in vitro cell-based assays. As shown in Fig. 3, cell proliferation, apoptosis, and cell cycle arrest were not changed by miR-153 overexpression in DLD-1 and HCT-116 cells with or without IFN-γ treatment (Fig. 3a, b). IFN-γ treatment increased cancer cell migration (Fig. 3c), but slightly decreased cell proliferation after 1 to 6 days (Fig. 3d), and decreased colony formation (Fig. 3e). However, miR-153 introduction into either HCT-116 or DLD-1 cells barely altered cell proliferation, cell migration, and colony formation. Previous reports demonstrate that miR-153 overexpression reduces cell proliferation in lung cancer cells [20], melanoma cells [21], and glioblastoma stem cells [22], but does not affect cell proliferation in colon cancer cells [23]. Our data demonstrate that overexpression of miR-153 and subsequent IDO1 inhibition do not alter cell proliferation and other cellular processes in colon cancer cells. These data suggest that miR-153-mediated cell proliferation regulation is cancer cell-type specific. It is notable that overexpression of miR-153 downregulated IDO1 expression about 50% in HCT-116 and DLD-1 cells (Fig. 2c), which did not affect cell proliferation, cell migration, and colony formation of these cells (Fig. 3). However, when IDO1 expression was completely depleted in HCT-116 and HT-29 cells, cell proliferation was significantly reduced [24]. These results imply that moderate downregulation of IDO1 by miR-153, but not elimination of IDO1, has little effect on colon cancer cells.

### Generation of EGFRvIII-CAR T cells targeting colon cancer

**Fig. 4:**
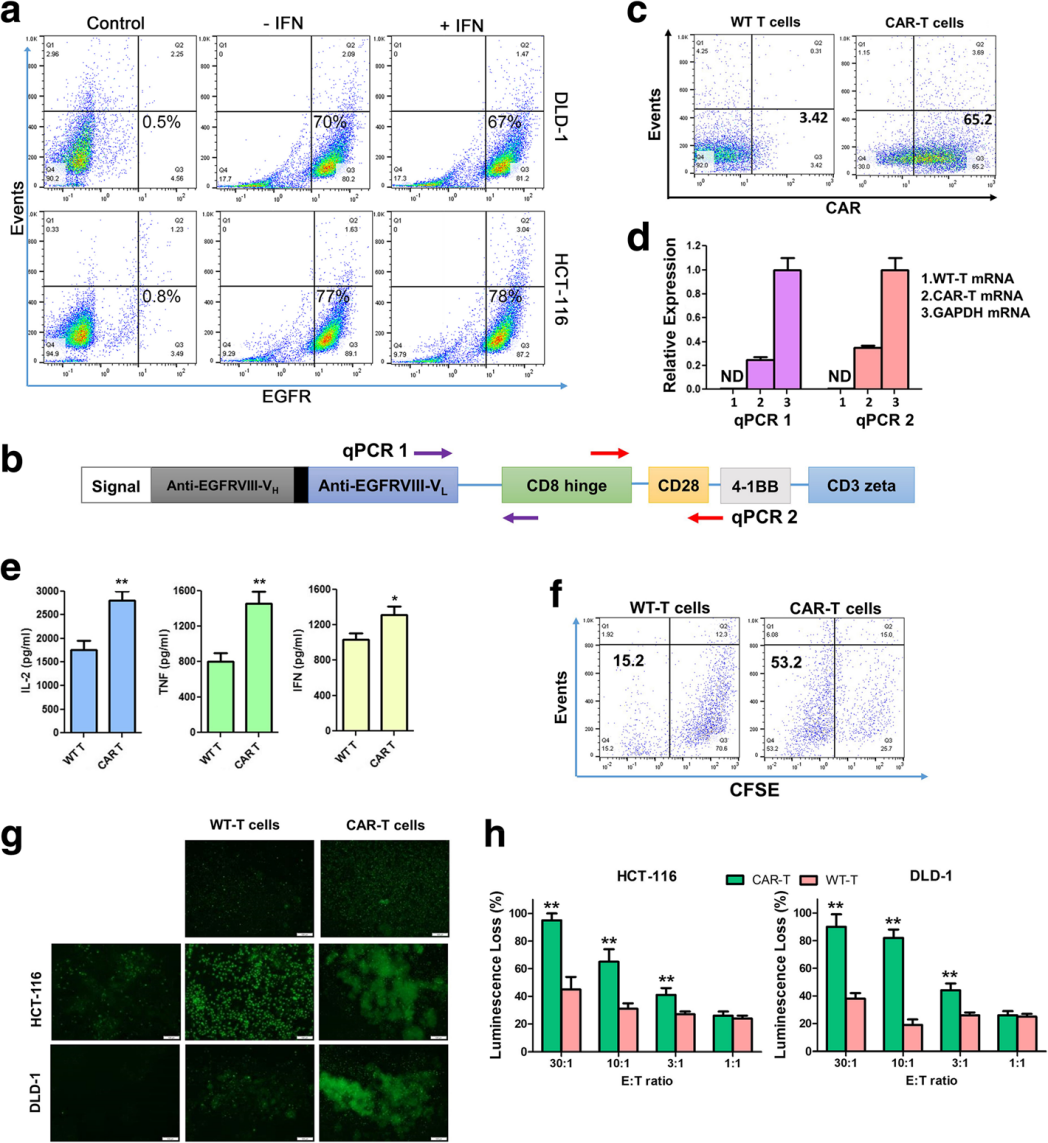
CAR T cells exhibit an increased cytotoxicity against colon cancer cells. **a** EGFR expression in DLD-1 and HCT-116 cells measured by flow cytometry. Cells were treated with 30 ng/ml IFN-γ for 24 h before incubated with antibodies against EGFR or an IgG control (control). **b** The EGFRvIII-CAR transgene structure. **c** Expression of EGFRvIII-CAR molecules in primary T cells as measured by flow cytometry. **d** qPCR analysis of EGFRvIII-CAR expression in T cells. qPCR was performed using two pairs of oligonucleotides. The mRNA levels of the CAR were determined using GAPDH as a reference. ND not detectable. **e** Cytokine production of WT T and CAR T cells co-cultured with HCT-116 cells as measured by ELISA (*n* = 3). **f** CFSE-based cell proliferation assays of WT and CAR T cells co-cultured with HCT-116 cells. *X*-axis is for the CFSE intensity; *Y*-axis is for the events based on side-scattered light (cell numbers). **g** Apoptosis analyses of HCT-116 or DLD-1 cells co-cultured with WT T or CAR T cells. Green florescence represents the release of caspase 3/7. **h** CAR T cell-mediated killing of tumor cells. HCT-116 or DLD-1 cells carrying firefly luciferase gene were co-cultured with WT or CAR T cells at different ratios (E:T effector T cells to target tumor cells). Luminescence was measured as a percentage of tumor cell death. The green line denotes CAR T cells, and orange denotes WT T cells. *P ≤ 0.05; **P ≤ 0.01

EGFR was expressed at a high level on the surface of DLD-1 and HCT-116 (Fig. 4a). We constructed a CAR targeting the variant III mutation of EGFR (EGFRvIII) in a lentiviral vector. The scFv against EGFRvIII was from cetuximab, a humanized monoclonal antibody against EGFRvIII, which has a *K*_*D*_ of 101 nM for EGFRvIII and 872 nM for WT EGFR [13]. Thus, this EGFRvIII-CAR can target colon cancer cells expressing WT EGFR, albeit at a low efficiency. The scFv was fused to a CD8α-derived hinge and transmembrane domain, both CD28 and 4-1BB co-stimulation domains linked to the CD3ζ signaling domain, as previously reported [13]. The GM-CSF leader sequence was used for efficient expression and localization of CAR molecules (Fig. 4b). We generated CAR T cells expressing EGFRvIII-CAR (CAR T) or control cells (WT T) that were transduced by virus carrying the parental vector as described [13]. We labeled CAR T or WT T cells with antibodies recognizing the CAR, and the EGFRvIII-CAR transgene expression was detected in CAR T cells but not WT T cells (Fig. 4c). qPCR analyses further detected the expression of the EGFRvIII-CAR mRNA in CAR T cells, but not in the WT T cells (Fig. 4d). Compared with WT T cells, CAR T cells released significantly more of the cytotoxic cytokines IFN-γ, TNF-α, and IL-2 (Fig. 4e) and had greater proliferation when they were co-cultured with HCT-116 cells (Fig. 4f). In addition, CAR T cells showed robust in vitro tumor cell killing activities when co-cultured with both HCT-116 and DLD-1 cells, as the co-cultured tumor cells released more caspase 3/7 proteins (Fig. 4g) and had greater cell lysis (Fig. 4h). Taken together, these results indicate that CAR T cells specifically recognize EGFR-positive tumor cells and enhance tumor cell apoptosis.

### miR-153 overexpression enhances cancer cell killing by CAR T cells in xenografts

**Fig. 5:**
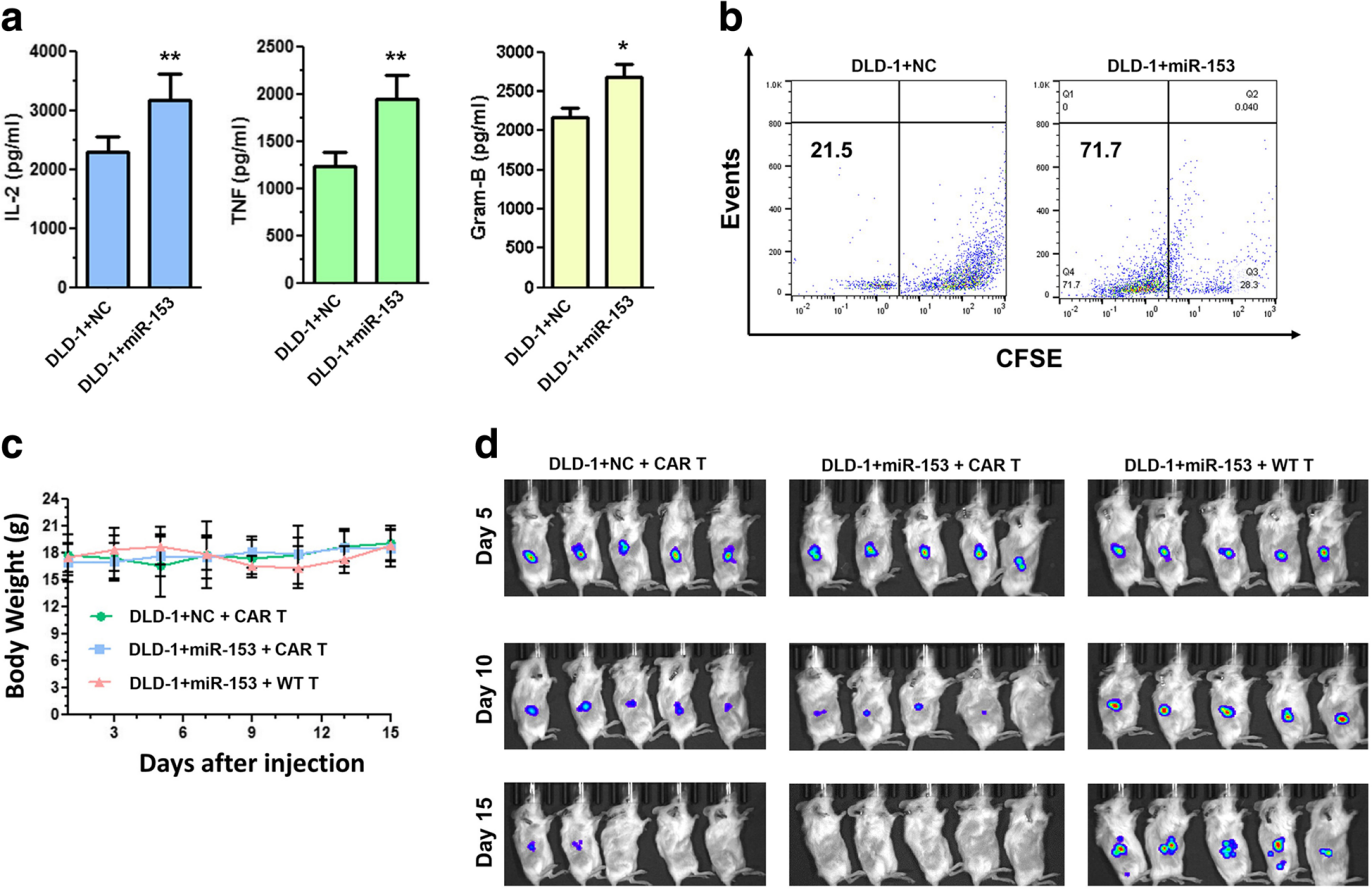
miR-153 overexpression within colon cancer cells enhances CAR T cells proliferation and attenuates tumor growth. **a** Cytokine production of CAR T cells co-cultured with DLD-1 cells with or without miR-153. Gram-B granzyme B. *P* ≤ 0.05 in all three. **b** CFSE-based proliferation of CAR T cells cultured with DLD-1 + NC or DLD-1 + miR-153. NC is a normal control for DLD-1-miR-153 (DLD-1-luc cells were infected with virus carrying the parental vector). **c** Body weight of NSG mice injected with tumor cells and T cells. **d** Xenograft tumor growth in NSG mice inoculated with DLD-1 + NC or DLD-1 + miR-153 cells and treated with CAR T or WT T cells. At 5 days after inoculation with tumor cells, CAR T cells or WT T cells (1 × 10^7^) were injected intravenously, and tumor growth was monitored with luciferase-based imaging every 5 days for 15 days. *P ≤ 0.05; **P ≤ 0.01

We next investigated potential anti-tumor benefit of CAR T cells when they encounter tumor cells overexpressing miR-153. A stable DLD-1 cell line overexpressing luciferase (DLD-1-luc) was transduced by a lentivirus carrying an expression cassette for miR-153, resulting in the DLD-1 + miR-153 line. A control cell line, DLD-1-NC, was obtained by infecting DLD-1-luc cells using a virus carrying the parental vector. We found that CAR T cells co-cultured with DLD-1 + miR-153 produced more IL-2, TNF-α, and granzyme B and had higher proliferation and potentially higher T cell activation than those co-cultured with DLD-1-NC (Fig. 5a, b). The expression of T cell activation markers, checkpoint markers, and co-stimulation markers was similar to each other (Additional file 5: Figure S5). These data indicate that IDO1 downregulation by miR-153 in colon cancer cells enhances the cytotoxicity of co-cultured CAR T cells. Our results are in agreement with a previous study in which higher expression of IL-2, TNF-α, and granzyme B in CAR T cells was also observed, when similarly made CAR T cells were co-cultured with target tumor cells with EGFR or EGFRvIII [13]. The underlying mechanism could be reduction of immunosuppression by IDO1.

We next injected DLD-1 + miR-153 and DLD-1 + NC cells into the flank of immunodeficient NSG mice (*n* = 5). Animal body weight was measured (Fig. 5c), and tumor growth was followed using in vivo bioluminescence monitoring (Fig. 5d). Mouse body weight within each group changed little during tumor cell engraftment and subsequent treatment with EGFRvIII-CAR T cells or control T cells (Fig. 5c). Tumor growth was similar between the two groups at day 5 after tumor cell inoculation, when we infused mice with CAR T cells or WT T cells via the tail vein. At day 10, tumors were smaller in mice injected with either DLD-1 + NC or DLD-1 + miR-153 treated with CAR T cells than those in mice with DLD-1 + miR-153 and WT T cells; mice with DLD-1 + miR-153 and CAR T injection had the smallest tumors. At day 15, tumors were eradicated in 5/5 mice with DLD-1 + miR-153 and EGFRvIII-CAR T and in three of five mice with mice with DLD-1 + NC and EGFRvIII-CAR T. Four of five mice with DLD-1 + miR-153 and WT T cells developed larger tumors, some of which had metastasized (Fig. 5d). These data support that autonomous overexpression of miR-153 within tumor cells enhanced anti-tumor activities of CAR T cells.

## Discussion

Tumor cells evade immunosurveillance through various mechanisms, including checkpoint inhibition of T cell activation and upregulation of IDO1 that catalyzes tryptophan degradation in the kynurenine pathway. Tryptophan depletion and its metabolite kynurenine create an immunosuppressive tumor microenvironment. Among the three IDO enzymes catabolizing tryptophan, IDO1 expression is limited in normal human tissues [25] but is high in tumor tissues [4], making it an ideal target for cancer therapy. Upregulation of IDO1 by IFN-γ in tumor cells is believed to generate an immunosuppressive tumor microenvironment that restricts cytotoxic T cells [4]. Recent clinical trials suggest that the IDO1 inhibitors indoximod and epacadostat are well tolerated by cancer patients and exert anti-cancer effects in a subset of patients [26–28]. To date, no IDO1 inhibitors are approved by the FDA to treat cancer. Limited evidence are available to support that CAR T cells are suppressed directly by IDO1. Yet, a recent study demonstrates that post infusion of CAR T cells, tumor specimens in glioblastoma patients have markedly increased expression of many immunosuppressive molecules, particularly IDO1 and FoxP3, and in some cases, IL-10, PD-L1, and/or TGF-β [29]. Among the three cancer immunotherapeutic approaches––PD-L1 blockade, CAR T, and IDO1 inhibition, combinations of two have been attempted: PD-L1 blockade enhances CAR T cell activity [30, 31], and the most recent trials testing an IDO1 inhibitor in combination with anti-PD-1 antibodies demonstrate promising syngeneic activity in multiple types of solid tumors [32]. However, the combination of CAR T and IDO1 inhibition has not been tested. In this work, we present that IDO1 inhibition by an miRNA miR-153 in combination with CAR T cells effectively enhances the efficacy of cancer cell killing that is mediated by CAR T cells.

We showed that IDO1 is induced by IFN-γ in colon cancer cells, and its expression is inversely correlated with survival in colon cancer patients. miR-153 downregulates IDO1 expression through targeting its 3′UTR. The expression of miR-153 is inversely correlated with IDO1 expression in colon tumors. miR-153 overexpression in colon cancer cells, however, does not alter cell proliferation, cell apoptosis, or colony formation in vitro. We measured the concentration of kynurenine in the medium of DLD-1 tumor cells co-cultured with CAR T cells, but found no differences between tumor cells with miR-153 and those without. There are two possible explanations. First, the change in kynurenine in the tumor microenvironment is so subtle that the differences cannot be adequately detected, yet is sufficient for reversing the tumor-suppressive environment. Second, IDO1 has an unknown alternative immunosuppressive role. We noted that indoximod, currently one of the most studied IDO inhibitors, does not inhibit tryptophan to kynurenine catabolism efficiently either [4].

We next construct a CAR against colon cancer cells using EGFR as a target. CAR T cell immunotherapy in colon cancer remains challenging, largely due to the lack of appropriate surface antigens whose expression is confined to malignant cells only [33, 34]. Guo et al. reported EGFR CAR T in patients with cholangiocarcinomas and gallbladder carcinomas [35]. Yet, it only achieved median progression-free survival of 4 months, and T cells exhaustion and non-specific toxicity were blamed for the less encouraging anti-tumor efficacy (as many tissues express EGFR at physiological conditions [36]). We use a “placeholder” CAR that is designed to target EGFRvIII, yet has restrained activity for EGFR. This CAR, based on the monoclonal antibody cetuximab, is less efficient in targeting EGFR-expressing tumor cells than EGFRvIII-positive cells [13]. Yet, it has been successfully used in preclinical models of glioblastoma, up to 30% of which have the mutant EGFRvIII [13]. Patients with a colorectal tumor bearing WT *KRAS*, which often expresses WT *EGFR*, do benefit from cetuximab therapy [37]. Nonetheless, CAR T cells expressing this CAR transgene demonstrate stronger tumoricidal activity toward WT EGFR-expressing colon cancer cells with miR-153 overexpression than they do toward the cells without miR-153 overexpression. When tested in immunodeficient mice, we show that CAR T cells completely eliminate colon cancer cells with miR-153 overexpression, but are only partially effective against colon cancer cells without miR-153 overexpression. Therefore, miR-153 and IDO1 are suitable adjunct drug targets, e.g., in combination with CAR T cells, for adequately unleashing the immune response against cancer.

## Conclusions

Our findings indicate that miR-153 inhibits IDO1 expression in colon cancer cells and is a tumor-suppressive miRNA that enhances CAR T cell immunotherapy. We expect that as solid tumor-specific CARs are developed, the combination of CAR T cells and IDO1 inhibition by miRNAs or other pharmacological approaches will be an effective treatment option for colorectal cancer and other solid tumors.

## Additional files

Additional file 1: **Figure S1.** IDO1 was induced by IFN-γ. IDO1 was induced by IFN-γ. (A) IDO1 expression of A549 and DLD-1 stimulated by 1-300 ng/ml IFN-γ for 24 h. IDO1 expression was measured using flow cytometry. (B) IDO1 expression in DLD-1 cells treated with 30 ng/ml IFN-γ for 3-48h. (C) IDO1 expression curves plotted from data in (A) and (B). (PDF 368 kb)

Additional file 2: **Figure S2.** IDO2 and TDO expression in relationship to survival of patients with colorectal cancer. (A) Overall survival of patients with colon cancer (n = 382) in the TCGA database. Patients were classified as having high or low IDO2 (left) or TDO (right) expression according the median level. (B) Survival in days according to IDO2 expression level of patients in Panel A. (PDF 266 kb)

Additional file 3: **Figure S3.** miRNAs are predicted to target the IDO1 3' UTR. (A) Schematic representation of the pmirGLO vector carrying two dual luciferase genes and the IDO1 3' UTR. (B and C) Dual luciferase assays from 293T cells (B) or Hela cells (C) transfected with miR-153 and the pmirGLO construct. (PDF 189 kb)

Additional file 4: **Figure S4.** IDO1 downregulation by miR-153 is inducer and time independence. (A and B) IDO1 expression in DLD-1 cells treated by Cp-G DNA or LPS was down-regulated by miR-153 as measured by flow cytometry (A) or by western blotting (B). DLD-1 cells were treated with LPS or cp-G DNA for 12 h. (C and D) The IDO1 protein (C) or mRNA (D) levels in DLD-1 and HCT-116 cells transfected with or without miR-153 before treated with LPS for 6 to 24 h. (PDF 309 kb)

Additional file 5: **Figure S5.** The activation of T cells exhibits no difference when co-cultured with tumor cells with or without miR-153. T cells were co-cultured in DLD-1+miR-153 (orange) or DLD-1+NC cells (green) for 24 hours. The expression of the designated T cell activation markers was measured by flow cytometry. (PDF 206 kb)

## Abbreviations

ALL: Acute lymphoblastic leukemia
APC: Allophycocyanin
ATCC: American Type Culture Collection
CAR: Chimeric antigen receptor
CTLA-4: Cytotoxic T lymphocyte antigen-4
DMEM: Dulbecco’s Modified Eagle Medium
EGFR: Epidermal growth factor receptor
ELISA: Enzyme-linked immunosorbent assay
ERBB2: Erb-b2 receptor tyrosine kinase 2
FACS: Fluorescence-activated cell sorting
FBS: Fetal bovine serum
FDA: Food and Drug Administration
FITC: Fluorescein isothiocyanate
GFP: Green fluorescence protein
GM-CSF: Granulocyte-macrophage colony-stimulating factor
IDO1: Indoleamine 2,3-dioxygenase 1
IFN-γ: Interferon-γ
MHC: Major histocompatibility complex
miR-153: MicroRNA 153
NC: Normal control
NSG: NOD scid gamma (NOD.*Cg-Prkdc*^scid^*Il2rg*^tm1Wjl^/SzJ)
PCR: Polymerase chain reaction
PD-1: Programmed death-1
PVDF: Polyvinylidene difluoride
RLU: Relative luminescence units
RPMI medium: Roswell Park Memorial Institute medium
scFv: Single-chain variable fragment
TCGA: The Cancer Genome Atlas
TDO: Tryptophan 2, 3-dioxygenase
WT: Wild-type

## References

[1] Fesnak AD, June CH, Levine BL (2016). Engineered T cells: the promise and challenges of cancer immunotherapy. Nat Rev Cancer.

[2] Topalian SL, Taube JM, Anders RA, Pardoll DM (2016). Mechanism-driven biomarkers to guide immune checkpoint blockade in cancer therapy. Nat Rev Cancer.

[3] Han X, Bryson PD, Zhao Y, Cinay GE, Li S, Guo Y, Siriwon N, Wang P (2017). Masked chimeric antigen receptor for tumor-specific activation. Mol Ther.

[4] Zhai L, Spranger S, Binder DC, Gritsina G, Lauing KL, Giles FJ, Wainwright DA (2015). Molecular pathways: targeting IDO1 and other tryptophan dioxygenases for cancer immunotherapy. Clin Cancer Res.

[5] Hennequart M, Pilotte L, Cane S, Hoffmann D, Stroobant V, Plaen E, Eynde B (2017). Constitutive IDO1 expression in human tumors is driven by cyclooxygenase-2 and mediates intrinsic immune resistance. Cancer Immunol Res.

[6] Li F, Zhang R, Li S, Liu J (2017). IDO1: an important immunotherapy target in cancer treatment. Int Immunopharmacol.

[7] Brandacher G, Perathoner A, Ladurner R, Schneeberger S, Obrist P, Winkler C, Werner ER, Werner-Felmayer G, Weiss HG, Gobel G, Margreiter R, Konigsrainer A, Fuchs D, Amberger A (2006). Prognostic value of indoleamine 2,3-dioxygenase expression in colorectal cancer: effect on tumor-infiltrating T cells. Clin Cancer Res.

[8] Ino K, Yamamoto E, Shibata K, Kajiyama H, Yoshida N, Terauchi M, Nawa A, Nagasaka T, Takikawa O, Kikkawa F (2008). Inverse correlation between tumoral indoleamine 2,3-dioxygenase expression and tumor-infiltrating lymphocytes in endometrial cancer: its association with disease progression and survival. Clin Cancer Res.

[9] Godin-Ethier J, Hanafi LA, Piccirillo CA, Lapointe R (2011). Indoleamine 2,3-dioxygenase expression in human cancers: clinical and immunologic perspectives. Clin Cancer Res.

[10] Platanias LC (2005). Mechanisms of type-I- and type-II-interferon-mediated signalling. Nat Rev Immunol.

[11] Spranger S, Spaapen RM, Zha Y, Williams J, Meng Y, Ha TT, Gajewski TF (2013). Up-regulation of PD-L1, IDO, and T(regs) in the melanoma tumor microenvironment is driven by CD8(+) T cells. Sci Transl Med.

[12] Zhao XS, Wang YN, Lv M, Kong Y, Luo HX, Ye XY, Wu Q, Zhao TF, Hu YH, Zhang HY, Huo MR, Wan J, Huang XJ (2016). miR-153-3p, a new bio-target, is involved in the pathogenesis of acute graft-versus-host disease via inhibition of indoleamine- 2,3-dioxygenase. Oncotarget.

[13] Johnson LA, Scholler J, Ohkuri T, Kosaka A, Patel PR, McGettigan SE, Nace AK, Dentchev T, Thekkat P, Loew A, Boesteanu AC, Cogdill AP, Chen T, Fraietta JA, Kloss CC, Posey AD, Engels B, Singh R, Ezell T, Idamakanti N, Ramones MH, Li N, Zhou L, Plesa G, Seykora JT, Okada H, June CH, Brogdon JL, Maus MV (2015). Rational development and characterization of humanized anti–EGFR variant III chimeric antigen receptor T cells for glioblastoma. Sci Transl Med.

[14] Ren J, Liu X, Fang C, Jiang S, June CH, Zhao Y (2017). Multiplex genome editing to generate universal CAR T cells resistant to PD1 inhibition. Clin Cancer Res.

[15] Yuan P, He XH, Rong YF, Cao J, Li Y, Hu YP, Liu Y, Li D, Lou W, Liu MF (2017). KRAS/NF-kappaB/YY1/miR-489 signaling Axis controls pancreatic cancer metastasis. Cancer Res.

[16] He XH, Zhu W, Yuan P, Jiang S, Li D, Zhang HW, Liu MF (2016). miR-155 downregulates ErbB2 and suppresses ErbB2-induced malignant transformation of breast epithelial cells. Oncogene.

[17] Lu Z, Li Y, Takwi A, Li B, Zhang J, Conklin DJ, Young KH, Martin R, Li Y (2011). miR-301a as an NF-kB activator in pancreatic cancer cells. EMBO J.

[18] Grimson A, Farh K, Johnston W, Garrett-Engele P, Lim L, Bartel D (2007). MicroRNA targeting specificity in mammals: determinants beyond seed pairing. Mol Cell.

[19] Baek D, Villen J, Shin C, Camargo FD, Gygi SP, Bartel DP (2008). The impact of microRNAs on protein output. Nature.

[20] Yuan Y, Du W, Wang Y, Xu C, Wang J, Zhang Y, Wang H, Ju J, Zhao L, Wang Z, Lu Y, Cai B, Pan Z (2015). Suppression of AKT expression by miR-153 produced anti-tumor activity in lung cancer. Int J Cancer.

[21] Zeng HF, Yan S, Wu SF (2017). MicroRNA-153-3p suppress cell proliferation and invasion by targeting SNAI1 in melanoma. Biochem Biophys Res Commun.

[22] Zhao S, Deng Y, Liu Y, Chen X, Yang G, Mu Y, Zhang D, Kang J, Wu Z (2013). MicroRNA-153 is tumor suppressive in glioblastoma stem cells. Mol Biol Rep.

[23] Zhang L, Pickard K, Jenei V, Bullock MD, Bruce A, Mitter R, Kelly G, Paraskeva C, Strefford J, Primrose J, Thomas GJ, Packham G, Mirnezami AH (2013). miR-153 supports colorectal cancer progression via pleiotropic effects that enhance invasion and chemotherapeutic resistance. Cancer Res.

[24] Thaker AI, Rao MS, Bishnupuri KS, Kerr TA, Foster L, Marinshaw JM, Newberry RD, Stenson WF, Ciorba MA (2013). IDO1 metabolites activate beta-catenin signaling to promote cancer cell proliferation and colon tumorigenesis in mice. Gastroenterology.

[25] Theate I, van Baren N, Pilotte L, Moulin P, Larrieu P, Renauld JC, Herve C, Gutierrez-Roelens I, Marbaix E, Sempoux C, Van den Eynde BJ (2015). Extensive profiling of the expression of the indoleamine 2,3-dioxygenase 1 protein in normal and tumoral human tissues. Cancer Immunol Res.

[26] Soliman HH, Jackson E, Neuger T, Dees EC, Harvey RD, Han H, Ismail-Khan R, Minton S, Vahanian NN, Link C, Sullivan DM, Antonia S (2014). A first in man phase I trial of the oral immunomodulator, indoximod, combined with docetaxel in patients with metastatic solid tumors. Oncotarget.

[27] Soliman HH, Minton SE, Han HS, Ismail-Khan R, Neuger A, Khambati F, Noyes D, Lush R, Chiappori AA, Roberts JD, Link C, Vahanian NN, Mautino M, Streicher H, Sullivan DM, Antonia SJ (2016). A phase I study of indoximod in patients with advanced malignancies. Oncotarget.

[28] Kristeleit R, Davidenko I, Shirinkin V, El-Khouly F, Bondarenko I, Goodheart MJ, Gorbunova V, Penning CA, Shi JG, Liu X, Newton RC, Zhao Y, Maleski J, Leopold L, Schilder RJ (2017). A randomised, open-label, phase 2 study of the IDO1 inhibitor epacadostat (INCB024360) versus tamoxifen as therapy for biochemically recurrent (CA-125 relapse)-only epithelial ovarian cancer, primary peritoneal carcinoma, or fallopian tube cancer. Gynecol Oncol.

[29] D.M. O'Rourke, M.P. Nasrallah, A. Desai, J.J. Melenhorst, K. Mansfield, J.J.D. Morrissette, M. Martinez-Lage, S. Brem, E. Maloney, A. Shen, R. Isaacs, S. Mohan, G. Plesa, S.F. Lacey, J.M. Navenot, Z. Zheng, B.L. Levine, H. Okada, C.H. June, J.L. Brogdon, M.V. Maus, A single dose of peripherally infused EGFRvIII-directed CAR T cells mediates antigen loss and induces adaptive resistance in patients with recurrent glioblastoma, Science Translational Medicine 9 (2017). eaaa0984. 10.1126/scitranslmed.aaa0984 PMC5762203 28724573

[30] Rafiq S, Jackson HJ, Yeku O, Purdon TJ, van Leeuwen DG, Curran KJ, Ahmed RN, Cullen GD, Yan S, Wang P, Xiang J, Liu C, Brentjens RJ (2017). Enhancing CAR T cell anti-tumor efficacy through secreted single chain variable fragment (scFv) immune checkpoint blockade. Blood.

[31] Serganova I, Moroz E, Cohen I, Moroz M, Mane M, Zurita J, Shenker L, Ponomarev V, Blasberg R (2017). Enhancement of PSMA-directed CAR adoptive immunotherapy by PD-1/PD-L1 blockade. Mol Ther Oncol.

[32] Chism DD (2017). Urothelial carcinoma of the bladder and the rise of immunotherapy. J Natl Compr Cancer Netw.

[33] Yu S, Li A, Liu Q, Li T, Yuan X, Han X, Wu K (2017). Chimeric antigen receptor T cells: a novel therapy for solid tumors. J Hematol Oncol.

[34] Yazdanifar M, Zhou R, Mukherjee P (2016). Emerging immunotherapeutics in adenocarcinomas: a focus on CAR-T cells. Curr Trends Immunol.

[35] Guo Y, Feng KC, Liu Y, Wu Z, Dai H, Yang QM, Wang Y, Jia H, Han W. Phase I study of chimeric antigen receptor modified T cells in patients with EGFR-positive advanced biliary tract cancers. Clin Cancer Res. 2018;24:1277-86. 10.1158/1078-0432.CCR-17-0432 29138340

[36] Chen J, Zeng F, Forrester SJ, Eguchi S, Zhang M-Z, Harris RC (2016). Expression and function of the epidermal growth factor receptor in physiology and disease. Physiol Rev.

[37] Karapetis CS, Khambata-Ford S, Jonker DJ, O'Callaghan CJ, Tu D, Tebbutt NC, Simes RJ, Chalchal H, Shapiro JD, Robitaille S, Price TJ, Shepherd L, Au H-J, Langer C, Moore MJ, Zalcberg JR (2008). *K-ras* mutations and benefit from cetuximab in advanced colorectal cancer. N Engl J Med.

