# Correction to: miR-153 suppresses IDO1 expression and enhances CAR T cell immunotherapy

**DOI:** 10.1186/s13045-018-0633-1

**Published:** 2018-07-03

**Authors:** Qian Huang, Jiajia Xi, Lei Wang, Xu Wang, Xiaodong Ma, Qipan Deng, Yong Lu, Munish Kumar, Zhiyuan Zhou, Ling Li, Zhaoyang Zeng, Ken H. Young, Qing Yi, Mingzhi Zhang, Yong Li

**Affiliations:** 1 Department of Cancer Biology, Lerner Research Institute, Cleveland Clinic, Cleveland, OH 44195 USA; 2 Institute for Brain Research and Rehabilitation, South China Normal University, Guangzhou, 510631 China; 3 Raman Fellow (UGC), Department of Biochemistry, University of Allahabad, Allahabad, India; 4 Department of Oncology, The First Affiliated Hospital of Zhengzhou University, Lymphoma Diagnosis and Treatment Center of Henan Province, Zhengzhou, 450000 Henan Province China; 5 Cancer Research Institute, Central South University, Changsha, 410078 China; 6 Department of Hematopathology, The University of Texas M.D. Anderson Cancer Center, Houston, TX 77030 USA

## Correction

The original article [1] contains a spelling error in the authorship; the authors would like to note the correct spelling of the second author, Jiajia Xi.
