# Supplementary material for: miR-153 suppresses IDO1 expression and enhances CAR T cell immunotherapy
Source: J Hematol Oncol. 2018 Apr 23;11:58. doi: 10.1186/s13045-018-0600-x (PMC5914051; doi:10.1186/s13045-018-0600-x)
Supplement: Supplementary file 1 — Figure S1. IDO1 was induced by IFN-γ. IDO1 was induced by IFN-γ. (A) IDO1 expression of A549 and DLD-1 stimulated by 1-300 ng/ml IFN-γ for 24 h. IDO1 expression was measured using flow cytometry. (B) IDO1 expression in DLD-1 cells treated with 30 ng/ml IFN-γ for 3-48h. (C) IDO1 expression curves plotted from data in (A) and (B). (PDF 368 kb) [file 13045_2018_600_MOESM1_ESM.pdf]

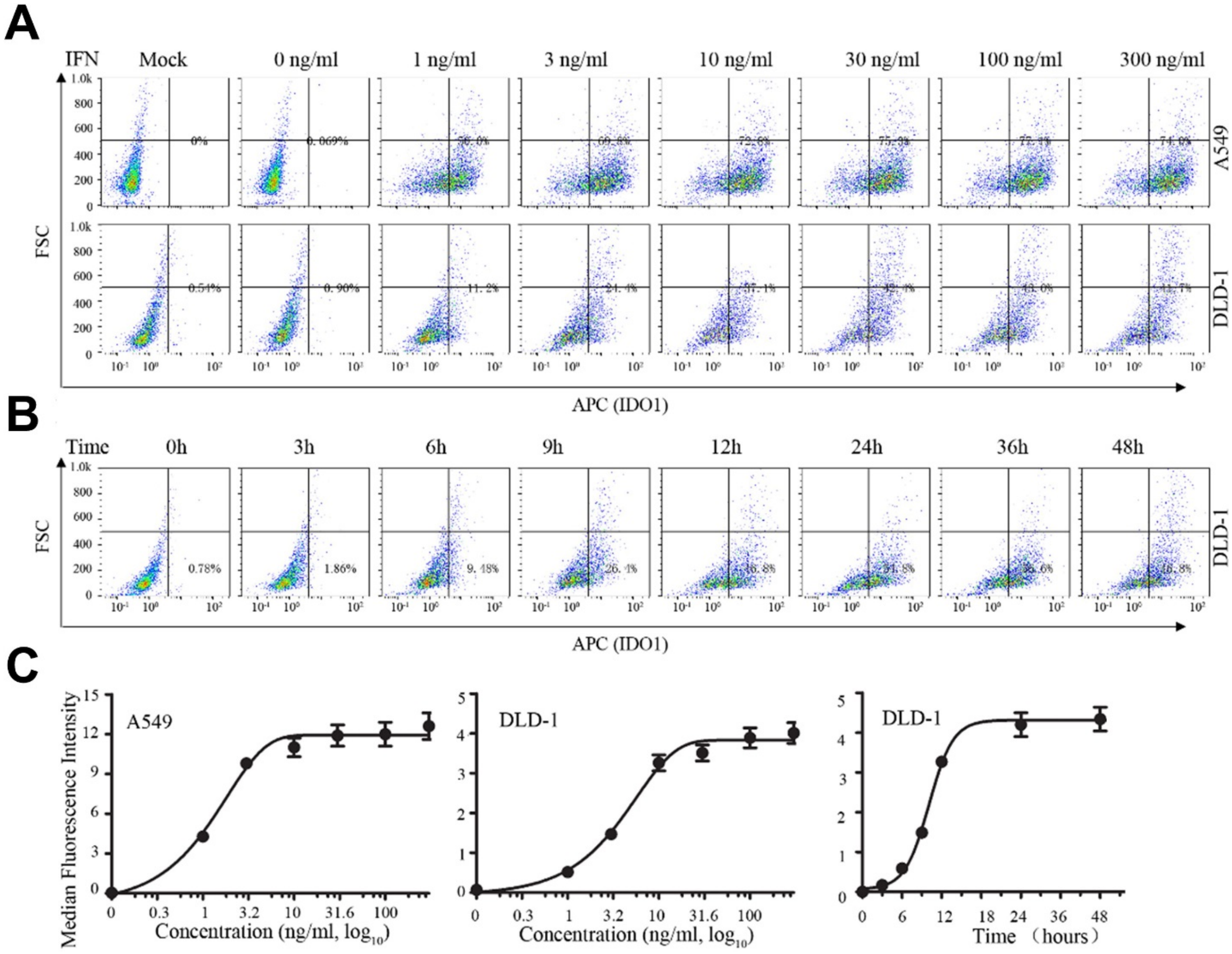

Figure S1. IDO1 was induced by IFN-γ. (A) IDO1 expression of A549 and DLD-1 stimulated by 1-300 ng/ml IFN-γ for 24 h. IDO1 expression was measured using flow cytometry. (B) IDO1 expression in DLD-1 cells treated with 30 ng/ml IFN-γ for 3-48h. (C) IDO1 expression curves plotted from data in (A) and (B).
