# Supplementary material for: miR-153 suppresses IDO1 expression and enhances CAR T cell immunotherapy
Source: J Hematol Oncol. 2018 Apr 23;11:58. doi: 10.1186/s13045-018-0600-x (PMC5914051; doi:10.1186/s13045-018-0600-x)
Supplement: Supplementary file 2 — Figure S2. IDO2 and TDO expression in relationship to survival of patients with colorectal cancer. (A) Overall survival of patients with colon cancer (n = 382) in the TCGA database. Patients were classified as having high or low IDO2 (left) or TDO (right) expression according the median level. (B) Survival in days according to IDO2 expression level of patients in Panel A. (PDF 266 kb) [file 13045_2018_600_MOESM2_ESM.pdf]

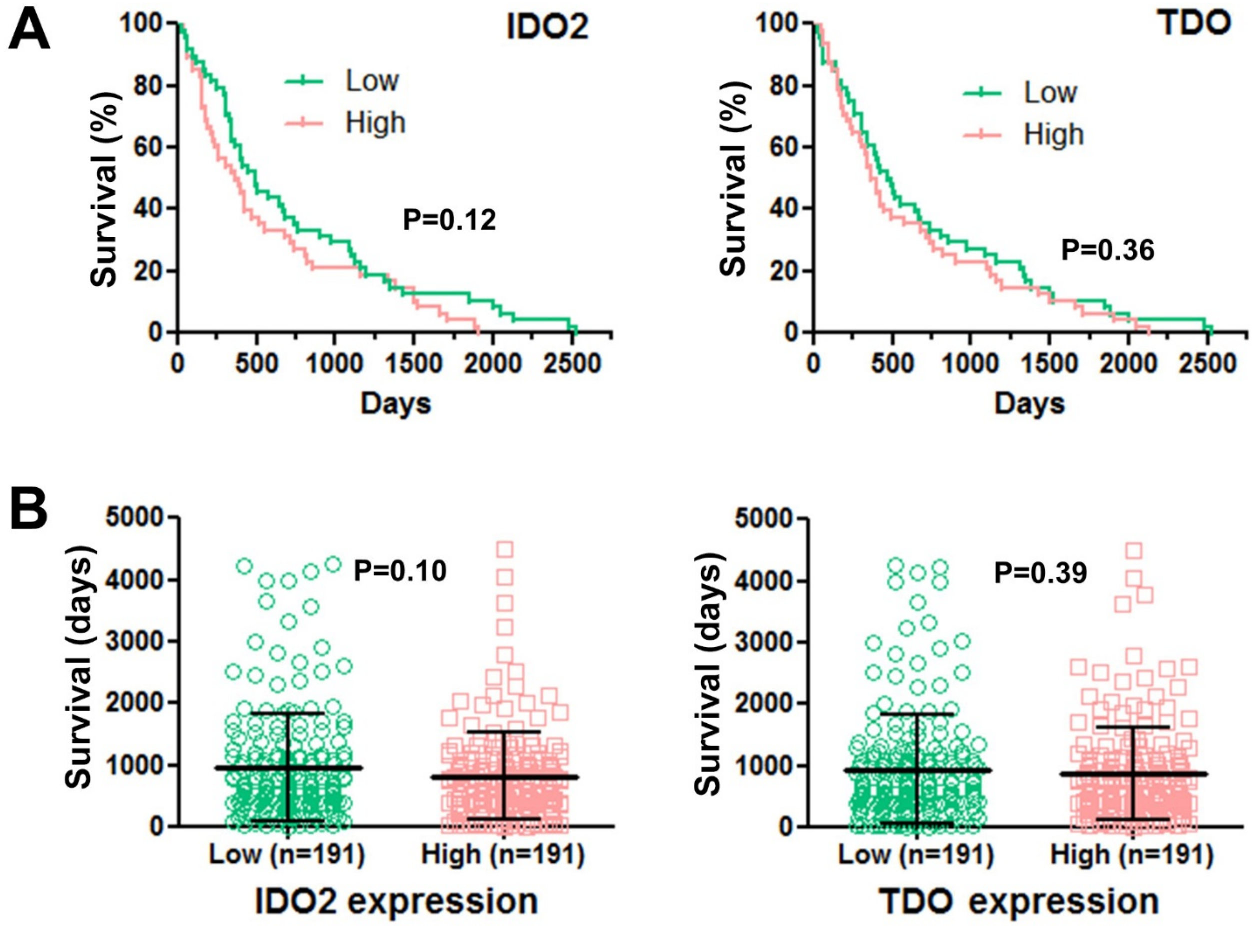

Figure S2. IDO2 and TDO expression in relationship to survival of patients with colorectal cancer. (A) Overall survival of patients with colon cancer (n = 382) in the TCGA database. Patients were classified as having high or low IDO2 (left) or TDO (right) expression according the median level. (B) Survival in days according to IDO2 expression level of patients in Panel A. Kaplan-Meier survival analysis followed by a log-rank test (A) and unpaired student's t-test (B) were performed.
