# Supplementary material for: miR-153 suppresses IDO1 expression and enhances CAR T cell immunotherapy
Source: J Hematol Oncol. 2018 Apr 23;11:58. doi: 10.1186/s13045-018-0600-x (PMC5914051; doi:10.1186/s13045-018-0600-x)
Supplement: Supplementary file 3 — Figure S3. miRNAs are predicted to target the IDO1 3' UTR. (A) Schematic representation of the pmirGLO vector carrying two dual luciferase genes and the IDO1 3' UTR. (B and C) Dual luciferase assays from 293T cells (B) or Hela cells (C) transfected with miR-153 and the pmirGLO construct. (PDF 189 kb) [file 13045_2018_600_MOESM3_ESM.pdf]

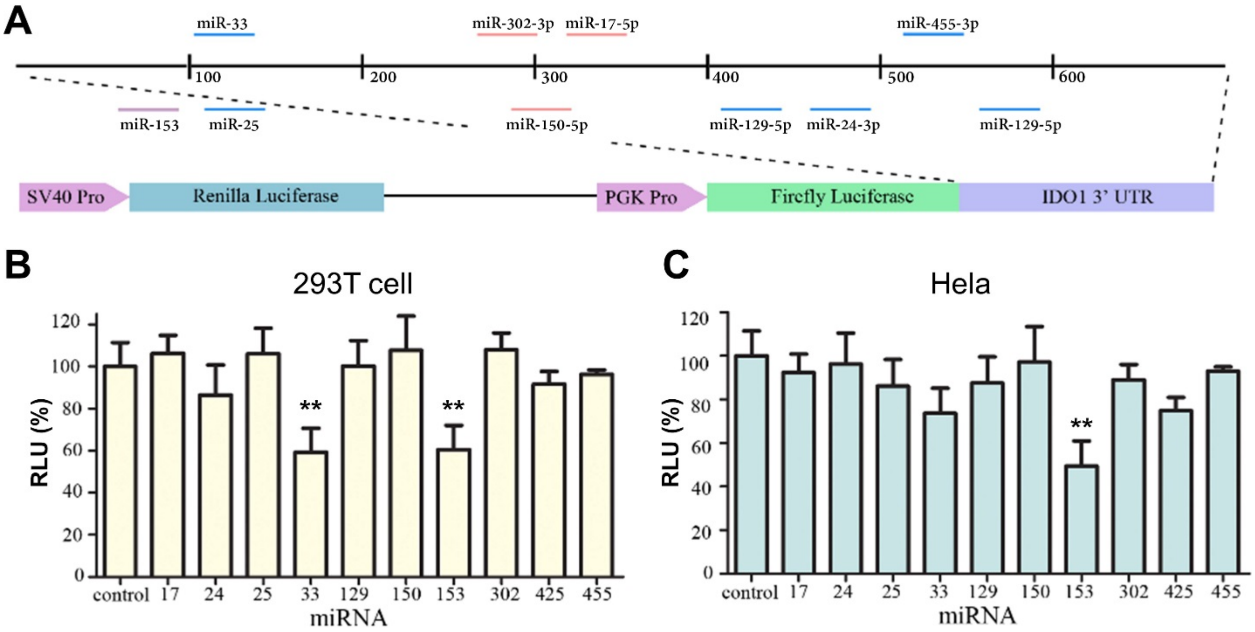

Figure S3. **miRNAs are predicted to target the IDO1 3' UTR**. (A) Schematic representation of the pmirGLO vector carrying two dual luciferase genes and the IDO1 3' UTR. (B and C) Dual luciferase assays from 293T cells (B) or Hela cells (C) transfected with miR-153 and the pmirGLO construct. Y axis denotes the relative luminescence units (RLU, firefly luciferase / Renilla luciferase) normalized to the miR-153 mimic control. Values denote mean ± SEM (n=3). **, $P \leq 0.01$.
