# Supplementary material for: miR-153 suppresses IDO1 expression and enhances CAR T cell immunotherapy
Source: J Hematol Oncol. 2018 Apr 23;11:58. doi: 10.1186/s13045-018-0600-x (PMC5914051; doi:10.1186/s13045-018-0600-x)
Supplement: Supplementary file 4 — Figure S4. IDO1 downregulation by miR-153 is inducer and time independence. (A and B) IDO1 expression in DLD-1 cells treated by Cp-G DNA or LPS was down-regulated by miR-153 as measured by flow cytometry (A) or by western blotting (B). DLD-1 cells were treated with LPS or cp-G DNA for 12 h. (C and D) The IDO1 protein (C) or mRNA (D) levels in DLD-1 and HCT-116 cells transfected with or without miR-153 before treated with LPS for 6 to 24 h. (PDF 309 kb) [file 13045_2018_600_MOESM4_ESM.pdf]

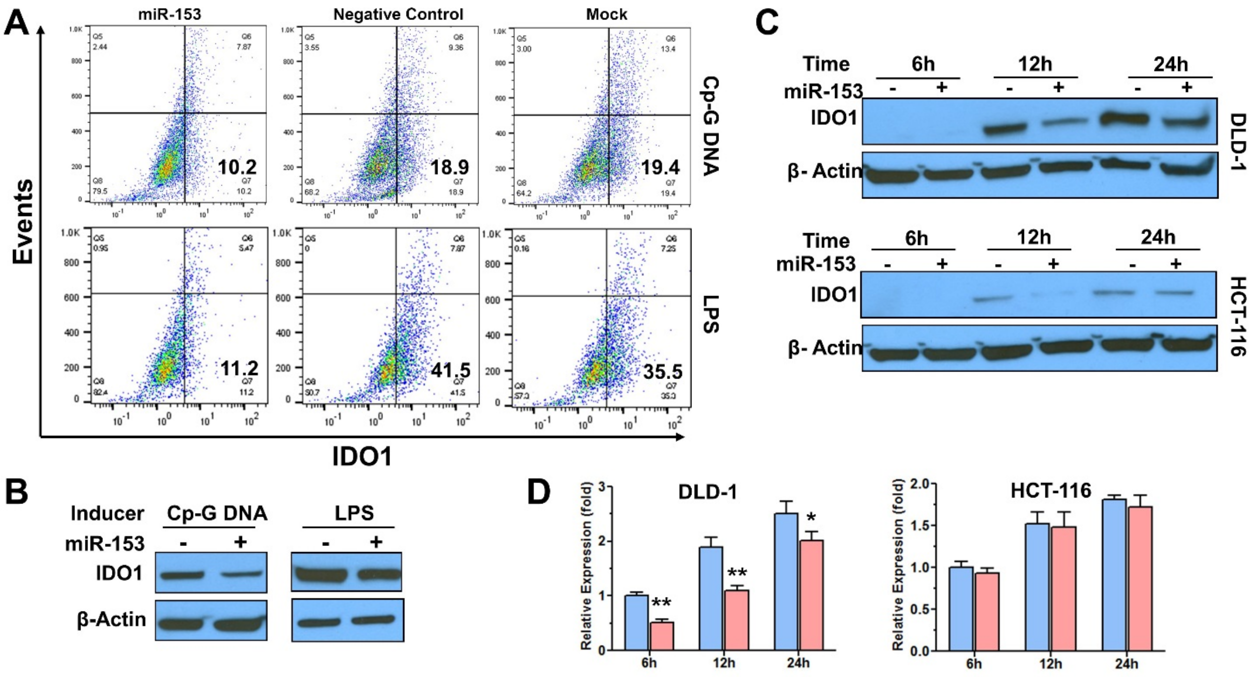

Figure S4. IDO1 downregulation by miR-153 is inducer and time independence. (A and B) IDO1 expression in DLD-1 cells treated by Cp-G DNA or LPS was down-regulated by miR-153 as measured by flow cytometry (A) or by western blotting (B). DLD-1 cells were treated with LPS or cp-G DNA for 12 h. (C and D) The IDO1 protein (C) or mRNA (D) levels in DLD-1 and HCT-116 cells transfected with or without miR-153 before treated with LPS for 6 to 24 h. *, P ≤0.05; **, P ≤ 0.01.
