# Supplementary material for: miR-153 suppresses IDO1 expression and enhances CAR T cell immunotherapy
Source: J Hematol Oncol. 2018 Apr 23;11:58. doi: 10.1186/s13045-018-0600-x (PMC5914051; doi:10.1186/s13045-018-0600-x)
Supplement: Supplementary file 5 — Figure S5. The activation of T cells exhibits no difference when co-cultured with tumor cells with or without miR-153. T cells were co-cultured in DLD-1+miR-153 (orange) or DLD-1+NC cells (green) for 24 hours. The expression of the designated T cell activation markers was measured by flow cytometry. (PDF 206 kb) [file 13045_2018_600_MOESM5_ESM.pdf]

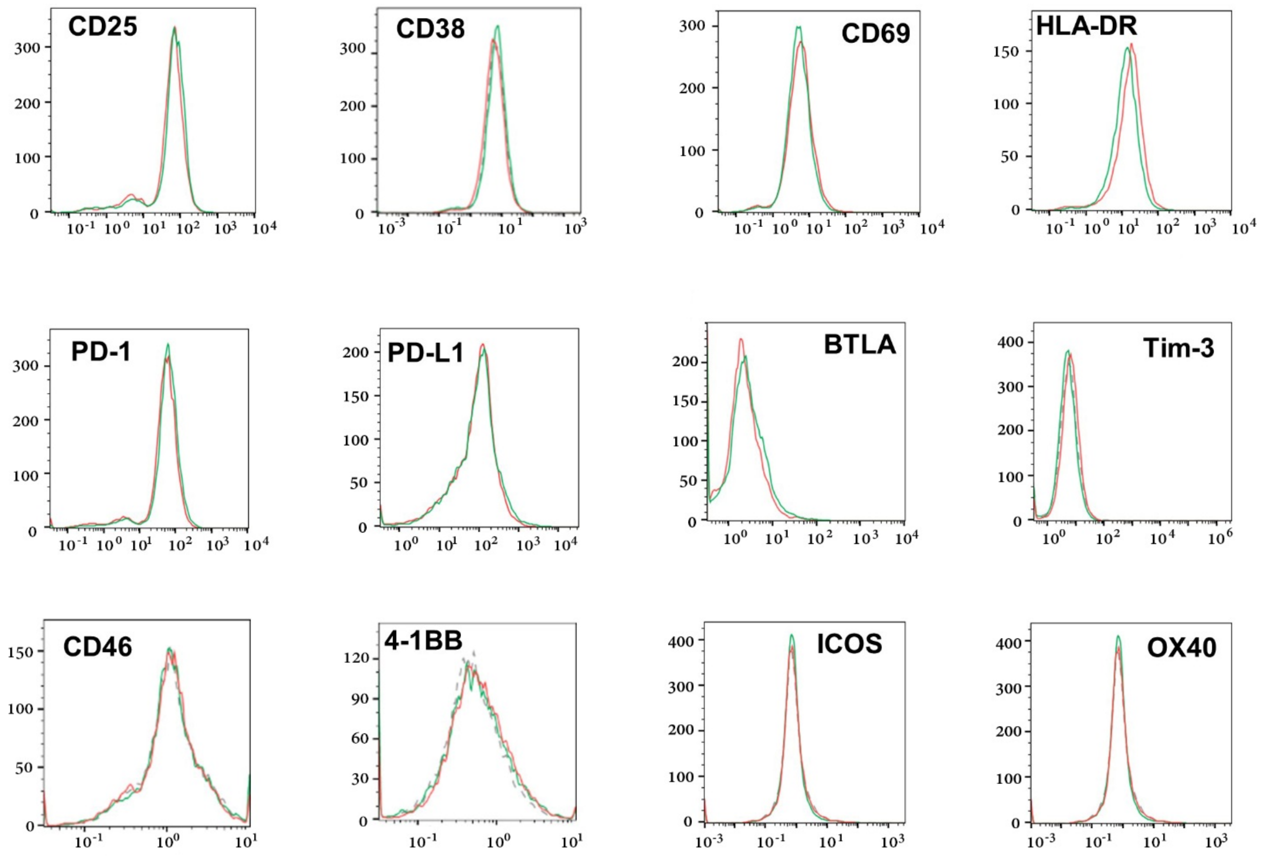


**Figure S5. The activation of T cells exhibits no difference when co-cultured with tumor cells with or without miR-153**. T cells were co-cultured in DLD-1+miR-153 (orange) or DLD-1+NC cells (green) for 24 hours. The expression of the designated T cell activation markers was measured by flow cytometry. Y axis denotes events (cell numbers); X axis denotes fluorescence density.
